# New Appliances and Things Medical

**Published:** 1902-12-06

**Authors:** 


### NEW APPLIANCES AND THINGS MEDICAL.

**[We shall be glad to receive at our Office, 28 & 29 Southampton Street, Strand, London, W.C., from the manufacturers, specimens of all new preparations and appliances which may be brought out from time to time.]**

### VISANUS. ROYAL HOSPITAL DISINFECTING FLUID AND VISANUS, DISINFECTING LIQUID SOAP.

(VISANUS COMPANY, 2 AND 4 CARRICK STREET, GLASGOW.)

"VISANUS" disinfecting fluid is a non-poisonous liquid of which we can speak highly as a disinfectant and deodoriser for general use. It is most powerfully antiseptic, non-corrosive, and does not burn or stain the skin. It therefore has the properties of a disinfectant which can safely be entrusted to the public and to uneducated persons for domestic use. The Visanus Royal Hospital disinfecting liquid soap is a preparation which is likely to become popular, since not only is it economical in use but it performs its work rapidly and effectively both as a cleaner and as a disinfectant. Since it contains no poisonous element it may be safely employed, and we commend it to the notice of sanitary authorities, and private persons who recognise the importance of keeping premises of all kinds in a sanitary and hygienic condition.

### COOK'S MEDICAL AND ANTISEPTIC SOAP.

(E. COOK AND CO., EAST LONDON SOAP WORKS, BOW, LONDON, E.)

WE have on several previous occasions called attention to Cook's high-class soaps, both for medical and domestic purposes. Cook's Biniodide Soap has long held the field as the most perfect and reliable antiseptic soap for the use of surgeons. Cook's Gold Medal Antiseptic Soap is a finely super-fatted preparation containing one of the most powerful germicides, especially suited for the disinfection of the dress, or any material which may have become infected with disease germs, and for washing hands and instruments before and after operation. These soaps, as the product of one of our most enterprising and thoroughly sound home industries, deserve the patronage of all patriotic Englishmen.

### LOFOTOL.

(SOUTHALL BROTHERS AND BARCLAY, LIMITED, MANUFACTURING CHEMISTS, BIRMINGHAM.

THE value of cod-liver oil in the treatment of wasting diseases and its extreme unpalatableness has encouraged the inventive genius of chemists to find either a vehicle for rendering the administration tolerable or some flavouring agent to disguise the taste. The latest device is the aëration of the oil with carbonic acid gas, and the product is called Lofotol, from the name of the island, Lofoten, where it is prepared and purified. Lofotol, then, is a sparkling oil, prepared from a pure cod-liver oil charged with carbonic acid gas. The advantages of this novelty in pharmaceutical art are:—1. Palatability, owing to the sharp sparkling taste. 2. Rapidity of digestion, owing to the stimulating influence of the gas. 3. Freedom from a tendency to turn rancid. Although the two latter claims require confirmation, there can be no doubt that, being charged with gas, the oil is far more pleasant to the palate than when taken without some adjuvant of the kind. Owing to the viscous character of the oil the gas does not readily escape and become "flat," nevertheless it must not be shaken or kept in a warm place or the effects of the aëration will be lost. We regard the method of preparation as very ingenious, and it is probable that Lofotol will become popular.

